# Serum Phosphate Predicts Early Mortality among Underweight Adults Starting ART in Zambia: A Novel Context for Refeeding Syndrome?

**DOI:** 10.1155/2013/545439

**Published:** 2013-04-16

**Authors:** John R. Koethe, Meridith Blevins, Christopher K. Nyirenda, Edmond K. Kabagambe, Janelle M. Chiasera, Bryan E. Shepherd, Isaac Zulu, Douglas C. Heimburger

**Affiliations:** ^1^Centre for Infectious Diseases Research in Zambia, Plot 1275 Lubuto Road, P.O. Box 34681, Lusaka 10101, Zambia; ^2^Division of Infectious Diseases, Department of Medicine, Vanderbilt University Medical Center, A2200-MCN, 1161 21st Avenue South, Nashville, TN 37232-2582, USA; ^3^Vanderbilt Institute for Global Health, 2525 West End Avenue, Suite 750, Nashville, TN 37203, USA; ^4^Department of Biostatistics, Vanderbilt University Medical Center, S2323 MCN, 1161 21st Avenue South, Nashville, TN 37232-2582, USA; ^5^University Teaching Hospital, Private Bag RWIX, Lusaka 10101, Zambia; ^6^Schools of Medicine and Public Health, University of Alabama at Birmingham, 1900 University Boulevard, Birmingham, AL 35294, USA

## Abstract

*Background*. Low body mass index (BMI) at antiretroviral therapy (ART) initiation is associated with early mortality, but the etiology is not well understood. We hypothesized that low pretreatment serum phosphate, a critical cellular metabolism intermediate primarily stored in skeletal muscle, may predict mortality within the first 12 weeks of ART. *Methods*. We prospectively studied 352 HIV-infected adults initiating ART in Lusaka, Zambia to estimate the odds of death for each 0.1 mmol/L decrease in baseline phosphate after adjusting for established predictors of mortality. *Results*. The distribution of phosphate values was similar across BMI categories (median value 1.2 mmol/L). Among the 145 participants with BMI <18.5 kg/m^2^, 28 (19%) died within 12 weeks. Lower pretreatment serum phosphate was associated with increased mortality (odds ratio (OR) 1.24 per 0.1 mmol/L decrement, 95% CI: 1.05 to 1.47; *P* = 0.01) after adjusting for sex, age, and CD4^+^ lymphocyte count. A similar relationship was not observed among participants with BMI ≥18.5 kg/m^2^ (OR 0.96, 95% CI: 0.76 to 1.21; *P* = 0.74). *Conclusions*. The association of low pretreatment serum phosphate level and early ART mortality among undernourished individuals may represent a variant of the refeeding syndrome. Further studies of cellular metabolism in this population are needed.

## 1. Introduction

Early mortality in the initial 90 days after antiretroviral therapy (ART) initiation is strikingly high among persons with low body mass index (BMI < 18.5 kg/m^2^) compared to those with normal BMI [1–4]. While a greater incidence of opportunistic infections and more advanced immunosuppression likely contributes to increased mortality in undernourished patients [5, 6], the loss of metabolically active tissue may negatively impact a range of critical physiologic processes and also contribute to these early deaths [7–9].

Skeletal muscle mass, a major reservoir of bioavailable phosphate, is reduced early in HIV-associated weight loss, and among HIV-uninfected persons chronic undernutrition is associated with a reduction in mitochondrial enzyme activity which rapidly normalizes with nutritional support [10–12]. Prior studies of malnourished prisoners-of-war and chronically ill hospital patients found that rapid depletion of serum phosphate after aggressive feeding disrupts electrolyte homeostasis and can induce cardiac and pulmonary complications and death—a condition termed *refeeding syndrome* [13–16]. We hypothesized that a similar syndrome resulting from increased metabolic demand for phosphate-dependent cellular metabolism intermediates (e.g., adenosine triphosphate and 2,3-diphosphoglycerate) after ART initiation, due to increased anabolism, physical activity, nutrient intake, viral suppression, or immune reconstitution, may deplete bioavailable phosphate stores in undernourished adults and contribute to the high early mortality observed in this population [17, 18]. To investigate whether hypophosphatemia is a novel determinant of early ART mortality, we measured pretreatment serum phosphate levels, several previously described predictors of poor survival, and vital status at 12 weeks among HIV-infected adults starting treatment with a range of BMI values in Lusaka, Zambia. 

## 2. Methods

Between November 2006 and December 2007, we enrolled HIV-infected adults starting ART at a single public sector primary care clinic in Lusaka, Zambia into two simultaneous observational cohorts to evaluate metabolic predictors of mortality within 12 weeks of treatment initiation. Both cohorts have been described previously [19–21]. Enrollment criteria were ART-eligibility according to national guidelines in place at the time (i.e., World Health Organization (WHO) stage 4 disease, a CD4^+^ lymphocyte count <200 cells/*μ*L, or WHO stage 3 disease and a CD4^+^ lymphocyte count <350 cells/*μ*L), intention to start therapy the same day and remain in the area for the study duration, and agreement to adhere to the additional study visits and laboratory testing requirements. One cohort (NEMART) [19] comprised 142 individuals with severe malnutrition and advanced disease (i.e., BMI <16 kg/m^2^ or CD4^+^ lymphocyte count <50 cells/*μ*L), and the other (DGPLEAD) [21], comprised 210 participants with BMI ≥16 kg/m^2^ and CD4^+^ ≥50 cells/*μ*L. All screened patients offered the opportunity to enroll in NEMART or DGPLEAD elected to participate in the study. In this analysis we combine data from both cohorts to evaluate the common primary study endpoint of all-cause mortality within the first 12 weeks of ART. 

Study participants were evaluated at enrollment by a research nurse and a clinical officer, and additional assessment was performed by a supervising physician as needed. The initial visit included a detailed health history, review of systems, physical examination, and laboratory testing (serum metabolic panel including electrolytes, phosphate, albumin, ferritin, and high-sensitivity C-reactive protein (hsCRP)). The initial ART regimen was selected from the national program formulary: lamivudine, efavirenz or nevirapine, and zidovudine or stavudine. In July 2007, tenofovir with emtricitabine replaced lamivudine, zidovudine, and stavudine in the national program's first-line regimen [22]. 

Participants in the NEMART cohort were evaluated by the study staff at 1, 2, 4, 8, and 12 weeks after initiating ART. Serum electrolyte measurements were done in real time, and when deficiencies of phosphate were detected participants were given supplements within seven days according to a predetermined algorithm based on serum levels. Participants in the DGPLEAD cohort were evaluated by study staff at baseline and after 12 weeks of ART; their serum samples were frozen at −80°F and assayed after the study was completed. 

CD4^+^ lymphocyte counts were performed using a Beckman Coulter Epics XL-MCL flow cytometer (Beckman Coulter, Inc., Fullerton, CA, USA). Chemistry assays were measured using a Roche COBAS Integra 400+ (Roche Diagnostics, Basel, Switzerland) or a Pointe 180 Chemistry Analyzer (Pointe Scientific, Canton, MI, USA). 

If a participant missed a study visit and could not be reached by mobile phone, community outreach teams attempted to locate the patient using housing locator forms completed at enrollment [23]. If the participant could not be located or credible information on vital status could not be obtained from relatives or the community, the participant was classified as lost to follow-up. 

We used logistic regression to assess the relationship between a 0.1 mmol/L decrease in baseline serum phosphate and the odds of death within 12 weeks of ART in the full cohort (including an interaction term for BMI and phosphate) and after stratifying the cohort according to WHO BMI criteria for grading malnutrition (<16, 16-17, and 17-18.49 kg/m^2^) [24]. All models were adjusted for sex, age, and CD4^+^ lymphocyte count at ART initiation and secondary analyses further adjusted for baseline albumin, ferritin, and hsCRP, and concomitant tuberculosis treatment. Patients lost to follow-up were excluded from primary analyses; a secondary analysis considered the combined endpoint of lost to follow-up or death. R-software 2.9.2 (http://www.r-project.org/) was used for data analyses.

The study protocol was approved by the University of Zambia Research Ethics Committee and the Institutional Review Boards at the University of Alabama at Birmingham and Vanderbilt University. All procedures were consistent with the ethical standards of the Helsinki Declaration of 1975, as revised in 2000. All participants provided written consent.

## 3. Results and Discussion

Vital status was available for 307 of 352 enrolled participants at 12 weeks (87%) and 45 were classified as lost to follow-up; 28 (19%) of those with pretreatment BMI <18.5 kg/m^2^ died compared to 12 (7%) of those with BMI ≥18.5 kg/m^2^. The alive, deceased, and lost to follow-up groups differed by median age, BMI, CD4^+^ lymphocyte count, and serum phosphate, ferritin, and hsCRP levels (Table 1; *P* < 0.05 for all comparisons). There was no difference between the three groups in the proportion of participants receiving tuberculosis treatment at the time of ART initiation. Among those with known vital status at 12 weeks, deceased participants had significantly lower BMI, CD4^+^ lymphocyte counts, serum albumin levels, and serum phosphate levels, but higher inflammation biomarkers (serum hsCRP and ferritin), compared to survivors (*P* < 0.01 for all comparisons).

Participants lost to follow-up prior to 12 weeks were more likely to be female and had a lower median age, but the clinical characteristics of this group did not consistently resemble either the alive or deceased participants. Lost participants had the lowest median CD4^+^ lymphocyte count (36 cells/*μ*L), which was closer to the median of 50 cells/*μ*L observed in the deceased group versus the 109 cells/*μ*L in the alive group. However, median serum levels of phosphate, albumin, ferritin, and hsCRP among the lost participants more closely approximated the alive group compared to the deceased. 

Baseline phosphate values were generally uniform across the BMI range (Spearman's rank correlation for BMI and phosphate: rho = −0.05, *P* = 0.41), indicating that low phosphate values were not clustered among the low-BMI participants (normal serum phosphate is 0.81–1.4 mmol/L) [25]. At week 12, 7 (8%) survivors and 5 (20%) deceased subjects in NEMART had received oral or intravenous phosphate; electrolyte repletion was not provided in DGPLEAD. There was no association between phosphate supplementation and mortality (*P* = 0.20). While tenofovir has been linked to renal phosphate wasting and toxicity in some reports, though not in randomized trials, the initiation of a tenofovir-containing regimen in our cohort was not associated with increased mortality (*P* = 0.12) [26–28].

In a logistic regression model excluding participants with unknown vital status and adjusted for sex, age, and CD4^+^ lymphocyte count, both serum phosphate (odds ratio (OR) 1.18 per 0.1 mmol/L decrease; *P* = 0.02) and BMI (OR 1.33 per 1.0 kg/m^2^ decrease; *P* < 0.01) were significantly associated with 12-week mortality. We did not detect an interaction of BMI and phosphate on the log-odds of mortality (*P* = 0.52). Participants with BMI <18.5 kg/m^2^ (*n* = 144; i.e., undernourished by WHO criteria) had an increased odds of morality (OR 1.24, 95% confidence interval [CI]: 1.05 to 1.47; *P* = 0.01) for each 0.1 mmol/L decrease in baseline phosphate. A similar relationship was observed among those with BMI <17 kg/m^2^ (*n* = 86, OR 1.25, 95% CI: 1.02 to 1.52; *P* = 0.03) (Table 2). Among those with BMI <16 kg/m^2^ (*n* = 49) the relationship between serum phosphate and outcome was not statistically significant (OR 1.06, 95% CI: 0.79 to 1.42; *P* = 0.70), though the death rate was substantially higher (27%). Among participants with BMI ≥18.5 kg/m^2^ (*n* = 161) we did not detect an association between baseline phosphate and 12-week mortality (OR 0.96, 95% CI: 0.76 to 1.21; *P* = 0.74). In a model including all patients but dichotomizing BMI at <18.5 versus ≥18.5, the interaction between BMI and phosphate was significant (*P* = 0.046).

When the logistic regression model was further adjusted for pretreatment serum hsCRP, ferritin, and albumin levels, there was minimal change in the odds of mortality with each 0.1 mmol/L reduction in baseline phosphate among those with BMI <18.5 kg/m^2^(OR 1.22, 95% CI: 1.01 to 1.48; *P* = 0.04). Similarly, the relationship between serum phosphate and mortality among those with BMI <18.5 kg/m^2^ remained significant after adjusting for concomitant tuberculosis treatment (OR 1.27, 95% CI: 1.06 to 1.51; *P* < 0.01), and TB treatment was not associated with mortality in the other models. 

When we repeated the analyses using the combined endpoint of death or loss to follow-up at 12 weeks, there was no significant association between a 0.1 mmol/L decrease in pretreatment serum phosphate and this outcome among all participants (OR 0.99, 95% CI: 0.90 to 1.08; *P* = 0.76) or those with a BMI <18.5 kg/m^2^ (OR 1.04, 95% CI: 0.93 to 1.17; *P* = 0.46). We attribute this lack of an association to likely heterogeneity in both the clinical characteristics and 12 week outcome among participants lost to follow-up.

Our study was limited by the inability to determine actual causes of death and to make definitive diagnoses of opportunistic infections (only local clinic facilities were utilized). Additionally, plasma HIV-1 viral load measurements were not utilized in routine clinical care at the time of the study, which may have introduced unmeasured confounding. The criteria for classifying a participant as lost to follow-up in our 12-week study differed from the definition used by the Zambian National ART program for pragmatic reasons. At the time of the study, patients in the national program were classified as lost to follow-up if they did not return to clinic within 37 days after a scheduled pharmacy visit or, if no pharmacy visit was scheduled, 60 days after the last clinical visit. This definition would not accurately classify participants at a single time point (i.e., 12 weeks), and therefore a more intensive outreach procedure utilizing home locator forms and phone calls was used to trace study participants. While some participants classified as lost may have eventually returned to care, the follow-up rates observed in our study approximated rates in the Zambian National ART program and in similar resource-limited settings [29].

We hypothesize that the observed association between lower pretreatment serum phosphate and early ART mortality may represent a variant of the refeeding syndrome associated with advanced HIV disease, in which depleted reserves of bioavailable phosphate (primarily skeletal muscle) are insufficient to maintain electrolyte homeostasis in response to a rise in phosphate-dependent cellular respiration after treatment initiation. The increased metabolic activity could result from viral suppression, immune reconstitution, or increased tissue repair and redeposition following a decline in inflammation-induced catabolism, possibly in combination with increased physical activity and dietary intake. Under these circumstances, low-BMI individuals with poor reserves of muscle mass may not be able to mobilize sufficient phosphate to meet metabolic demands. The consequences of a failure to maintain adequate bioavailable phosphate after ART initiation would likely mimic a classic refeeding syndrome, in which potentially lethal cardiovascular, respiratory, and neurologic sequelae result from electrolyte and fluid shifts accompany a reversal from catabolism and fat oxidation to the utilization of exogenous carbohydrate [14, 30–32]. 

At present, a role for refeeding syndrome in early ART mortality is provisional and additional studies are needed to understand whether the pathophysiologic processes contributing to death among malnourished individuals with low serum phosphate are consistent with this syndrome or a separate disorder. Further investigation of phosphate-dependent biological processes in these patients, including mitochondrial protein content and function, intracellular phosphate content, and potassium and magnesium homeostasis, will be critical for identifying the specific biological mechanisms involved and designing effective treatments to reduce mortality in the early ART period.

## 4. Conclusions

Low pretreatment serum phosphate levels were associated with increased risk for early mortality among adults with low BMI starting ART in Zambia. This association was independent of established risk factors for early ART mortality, including BMI, CD4^+^ lymphocyte count, serum albumin, and biomarkers of inflammation. The effect was not seen among individuals starting ART with normal BMI and may reflect an acute physiologic dysfunction resulting from insufficient bioavailable phosphate reserves in the immediate posttreatment period among individuals with reduced metabolically active tissue. Given the high early mortality rates among low BMI adults starting ART in Africa, further studies of metabolic dysfunction in this population are warranted to confirm these findings and explore potential mechanisms. Additionally, clinical trials to explore the benefits and risks of phosphate supplementation on ART outcomes among low-BMI individuals are needed.

## Figures and Tables

**Table 1:** Summary of baseline characteristics by 90-day outcome.

	Alive (*n* = 266)	Dead (*n* = 41)	Lost to follow-up (*n* = 45)	*P* value
Female sex: *n* (%)	151 (57%)	19 (46%)	30 (67%)	0.16
Age: years (IQR)	34 (29: 38)	35 (30: 40.2)	30 (26: 33)	<0.01
Body mass index: kg/m^2^	18.8 (17: 21)	16.7 (15.9: 18.6)	17 (15.8: 20.3)	<0.01
CD4^+^ lymphocyte count: cells/*µ*L	109 (57: 158)	50 (27: 129)	36 (22: 85)	<0.01
Phosphate (mmol/L)	1.3 (1.1: 1.4)	1.1 (0.9: 1.3)	1.3 (1.2: 1.5)	<0.01
Albumin (g/L)	31 (26: 35)	24 (20: 30)	32 (24: 35)	<0.01
Ferritin (mg/L)	207 (77: 506)	601 (313: 943)	209 (123: 487)	<0.01
C-reactive protein (mg/L)	5.5 (1.4: 21)	15.1 (6.7: 30)	4.6 (1.8: 20)	0.04
Receiving concomitant tuberculosis treatment (%)	61 (23%)	9 (22%)	10 (22%)	0.99
ART regimen: *n* (%)*				
AZT-3TC-EFV	9 (3%)	1 (3%)	1 (3%)	
AZT-3TC-NVP	96 (37%)	13 (33%)	6 (15%)	
D4T-3TC-EFV	13 (5%)	4 (10%)	1 (3%)	
D4T-3TC-NVP	94 (36%)	18 (46%)	13 (33%)	
TDF-FTC-EFV	3 (1%)	0	3 (8%)	
TDF-FTC-NVP	48 (18%)	3 (8%)	16 (40%)	
TDF-containing regimen: *n* (%)				0.120
TDF-based	51 (19%)	3 (8%)	19 (47%)	
Not TDF-based	212 (81%)	36 (92%)	21 (53%)	

Continuous variables are reported as medians (interquartile range). The distribution of study characteristics for participants by 90-day outcome is compared using the chi-square test: and continuous variables using the Wilcoxon rank-sum test.

*Ten participants were missing data on first ART regimen.

Abbreviations: ART: antiretroviral therapy; IQR: interquartile range; 3TC: lamivudine; d4T: stavudine; EFV: efavirenz; FTC: emtricitabine; NVP: nevirapine; TDF; tenofovir; ZDV: zidovudine.

**Table 2:** Pre-treatment serum phosphate and odds ratios of death prior to 12 weeks among study participants, stratified by BMI (*n* = 305)*.

BMI range	Patients at risk (deaths at 12 weeks)	Median pretreatment serum phosphate, mmol/L (IQR)	Odds ratio of mortality, per 0.1 mmol/L decrease in pre-treatment serum phosphate (95% CI)**	*P* value
All patients	305 (40)	1.2 (1.0, 1.4)	1.13 (1.00, 1.28)	0.06
≥18.5 kg/m^2^	161 (12)	1.2 (1.0, 1.4)	0.96 (0.76, 1.21)	0.74
<18.5 kg/m^2^	144 (28)	1.3 (1.1, 1.4)	1.24 (1.05, 1.47)	0.01
<17 kg/m^2^	86 (21)	1.3 (1.1, 1.4)	1.25 (1.02, 1.52)	0.03
<16 kg/m^2^	49 (13)	1.3 (1.1, 1.4)	1.06 (0.79, 1.42)	0.70

*Two patients with known vital status at 90 days (one alive and one deceased) were missing baseline BMI and excluded from the analysis.

**Adjusted for sex, age, and CD4^+^ lymphocyte count at ART initiation.

The effect of lower serum phosphate on the odds ratio of mortality remained significant for participants with BMI <18.5 kg/m^2^ when the model was further adjusted for pretreatment serum hsCRP, ferritin, and albumin levels (OR 1.22, 95% CI: 1.01 to 1.48; *P* = 0.04). When adjusted for concomitant tuberculosis treatment the odds ratio of mortality also remained significant for participants with BMI <18.5 kg/m^2^ (OR 1.27, 95% CI: 1.06 to 1.51; *P* < 0.01).
